# Supplementary material for: Blue Light Enhances the Antioxidant, Antimicrobial, and Antitumor Potential of the Green Microalgae Coelastrella sp. BGV
Source: Plants (Basel). 2024 Nov 23;13(23):3295. doi: 10.3390/plants13233295 (PMC11644280; doi:10.3390/plants13233295)
Supplement: Supplementary file 1 [file plants-13-03295-s001.zip › plants-3276772-supplementary.pdf]

Supplementary Material

Table S1. Comparison between the total phenolic and flavonoid content, and TAA of *Coelastrella* sp. BGV ethanol and aqueous extracts. The data are presented as mean ± SD (n ≥ 6). A one–way ANOVA (Holm–Sidak) test was applied to determine statistical differences between the variants, which are denoted by different letters ($p \leq 0.05$). The extracts were obtained from microalgae cultivated under white light.

**Solvent**	**Phenols** mg/g^{-1} **DW**	**Flavonoids** mg/g^{-1} **DW**	**TAA** mM/g^{-1} **DW**
Ethanol	43.08±0.001[a]	8.55±0.0005[a]	329.60±0.002[a]
Water	14.38±0.0005[b]	3.94±0.001[b]	80.42±0.0113[b]
